# Supplementary material for: Assessment of Phenotypic Characteristics, Polysaccharide Composition, and Hypoglycemic Potential in Different Commercial Grades of Lycium barbarum: A Comprehensive Study Using HPLC and NMR
Source: Foods. 2025 Nov 12;14(22):3862. doi: 10.3390/foods14223862 (PMC12651849; doi:10.3390/foods14223862)
Supplement: Supplementary file 1 [file foods-14-03862-s001.zip › foods---Supplementary Files.pdf]

Supplementary Table S1. The number of grains of different grades of *Lycium barbarum L.*

Number	Grades	Particle count（Particle/50g）	Number	Grades	Particle count（Particle/50g）
1	1	160	25	2	247
2	1	163	26	2	280
3	2	240	27	3	390
4	2	210	28	3	377
5	2	193	29	1	213
6	3	333	30	1	220
7	4	460	31	1	220
8	4	487	32	4	587
9	5	733	33	5	1083
10	1	153	34	5	890
11	2	290	35	3	313
12	2	377	36	3	383
13	3	363	37	4	493
14	3	370	38	4	583
15	3	410	39	4	550
16	2	373	40	5	897
17	4	617	41	5	783
18	1	273	42	5	927
19	4	607	43	5	830
20	1	240	44	1	187
21	1	233	45	2	230
22	3	303	46	3	353

Supplementary Table S2. The phenotypic characteristics of different grades of *Lycium barbarum L.*

Grades	Longitudinal diameter(mm)	diameter transversa(mm)	Average fruit weight per grain(g)	Unit weight(g)	Buoyancy rate(%)	*L**	*a**	*b**	*c*	*h*
1	13.86 ± 0.13	7.40 ± 0.09	0.22	0.50 ± 0.00	75.56 ± 4.16	33.00 ± 0.00	33.33 ± 0.47	32.00 ± 0.82	46.94 ± 0.38	47.02 ± 1.33
	15.82 ± 0.44	7.97 ±0.07	0.24	0.45 ± 0.00	98.89 ± 1.57	31.67 ± 1.25	39.00 ± 0.82	33.67 ± 1.25	51.54 ± 0.76	49.21 ± 1.85
	14.67 ±0.12	7.59 ± 0.08	0.23	0.47 ± 0.01	92.22 ± 1.57	31.67 ± 1.25	40.00 ± 3.27	33.67 ± 3.09	52.30 ± 5.20	49.93 ± 2.03
	14.14 ± 0.25	7.73 ± 0.10	0.23	0.48 ±0.00	91.11 ± 4.16	32.00 ± 0.00	36.00 ± 0.82	31.33 ± 1.25	47.73 ± 1.60	48.98 ± 1.06
	14.16 ± 0.41	7.39 ± 0.09	0.27	0.51 ± 0.00	91.11 ± 3.14	29.67 ± 0.47	35.33 ± 0.94	29.67 ± 1.25	46.14 ± 1.84	49.10 ± 0.65
2	14.28 ± 0.19	7.23 ± 0.03	0.19	0.46 ± 0.02	94.44 ± 1.57	33.00 ± 0.82	33.00 ± 0.82	34.33 ± 0.94	47.62 ± 1.47	43.87 ± 0.84
	13.69 ± 0.40	7.24 ± 0.05	0.2	0.47 ± 0.01	86.67 ± 2.72	32.00 ± 0.82	32.00 ± 0.82	32.33 ± 0.94	45.49 ± 1.47	44.71 ± 0.51
	13.72 ± 0.17	6.87 ± 0.22	0.18	0.46 ± 0.01	93.33 ± 4.71	33.33 ± 0.47	38.67 ± 0.47	38.67 ± 0.47	54.68 ± 0.82	45.00 ± 0.00
	13.14 ± 0.37	6.34 ± 0.08	0.17	0.51 ±0.01	70.00 ± 9.81	31.33 ± 1.70	42.33 ± 2.49	36.33 ± 2.63	55.81 ± 3.40	49.38 ± 1.99
3	13.20 ± 0.23	5.76 ± 0.10	0.11	0.44 ± 0.01	93.33 ± 2.72	33.67 ± 0.47	41.67 ± 0.47	35.00 ± 1.41	54.43 ± 0.96	49.99 ± 1.64
	12.69 ± 0.07	6.25 ±0.06	0.13	0.44 ± 0.01	98.89 ± 1.57	33.00 ± 0.82	40.00 ± 1.41	34.67 ± 0.47	52.95 ± 0.94	49.07 ± 1.68
	13.31 ± 0.51	6.22 ± 0.07	0.16	0.52 ± 0.00	70.00 ± 5.45	32.67 ± 0.47	35.67 ± 0.47	32.00 ± 0.82	47.92 ± 0.79	48.11 ± 1.01
	12.57 ± 0.28	6.01 ± 0.09	0.13	0.45 ± 0.00	95.56 ± 3.14	32.67 ± 1.25	40.67 ± 0.94	36.33 ± 1.89	54.54 ± 2.40	48.25 ± 0.98
4	11.65 ± 0.19	5.71 ± 0.12	0.12	0.52 ± 0.01	67.78 ± 8.75	31.33 ± 0.47	31.33 ± 0.47	31.00 ± 1.41	44.09 ± 1.09	45.33 ± 1.89
	10.93 ± 0.06	5.04 ± 0.08	0.09	0.47 ± 0.01	86.67 ± 4.71	34.00 ± 0.00	38.33 ± 0.47	35.33 ± 0.94	52.14 ± 0.35	47.34 ± 1.37
	12.77 ± 0.21	5.21 ± 0.09	0.1	0.43 ± 0.01	96.67 ± 2.72	33.33 ± 0.74	41.67 ± 1.70	36.33 ± 1.89	55.29 ± 3.06	48.93 ± 0.54
	11.57 ± 0.18	5.05 ± 0.08	0.09	0.43 ± 0.01	97.78 ± 1.57	34.00 ± 0.82	42.33 ± 0.47	37.33 ± 0.94	56.45 ± 0.32	48.60 ± 1.27
	11.75 ± 0.09	5.18 ± 0.06	0.09	0.42 ± 0.01	98.89 ± 1.57	34.33 ± 0.47	40.67 ± 0.94	36.33 ± 0.94	54.55 ± 0.11	48.22 ± 1.71
5	9.13 ±0.14	3.79 ± 0.06	0.05	0.51 ± 0.00	55.56 ± 4.16	34.00 ± 0.00	38.33 ± 0.47	35.00 ± 1.41	51.92 ± 1.42	47.62 ± 1.27
	10.95 ± 0.24	4.11 ± 0.07	0.06	0.44 ± 0.01	92.22 ±1.57	33.67 ± 0.47	41.67 ± 0.94	38.00 ± 1.41	56.39 ± 2.02	47.67 ± 0.50
	10.22 ± 0.17	4.01 ± 0.08	0.06	0.45 ± 0.01	93.33 ± 4.71	32.67 ± 1.25	44.00 ± 0.82	39.33 ± 1.25	59.02 ± 1.76	48.21 ± 0.48
	11.14 ± 0.03	4.36 ± 0.07	0.06	0.45 ± 0.01	82.22 ± 4.16	33.00 ± 1.00	40.50 ± 0.50	36.00 ± 1.00	54.20 ± 0.41	48.37 ± 1.61
	8.86 ± 0.08	4.16 ± 0.12	0.05	0.47 ± 0.01	75.56 ± 8.31	32.50 ± 0.50	39.00 ± 0.00	34.50 ± 1.50	52.08 ± 1.41	48.53 ± 1.75
	11.12 ± 0.02	4.37 ± 0.05	0.06	0.43 ± 0.00	100.00 ± 0.00	34.50 ± 1.50	42.00 ± 2.00	40.50 ± 1.50	58.35 ± 3.51	46.03 ± 0.43
